# Recommendations for Regulating the Environmental Risk of Shedding for Gene Therapy and Oncolytic Viruses in Canada

**DOI:** 10.3389/fmed.2019.00058

**Published:** 2019-03-28

**Authors:** Tania Bubela, Ron Boch, Sowmya Viswanathan

**Affiliations:** ^1^Faculty of Health Sciences, Simon Fraser University, Burnaby, BC, Canada; ^2^BIOTECanada, Ottawa, ON, Canada; ^3^Arthritis Program, Krembil Research Institute, University Health Network, Toronto, ON, Canada; ^4^Cell Therapy Program, University Health Network, Toronto, ON, Canada; ^5^Institute of Biomaterials and Biomedical Engineering, University of Toronto, Toronto, ON, Canada; ^6^Division of Hematology, Department of Medicine, University of Toronto, Toronto, ON, Canada

**Keywords:** environmental concerns, risk assessment, streamlined review, regulatory burden, clinical trials, immunotherapy

## Abstract

Canadian academic and industry stakeholders are concerned about the inclusion of “virus-like particles or sub-viral particles” in the definition of *New Substances Notification Regulations for Organisms (NSNR(O))* which impacts clinical cell and gene therapy and commercialization. The requirement of an independent 120 days Environment and Climate Change Canada (ECCC) review preceding a Health Canada review on quality *and* environmental concerns places an additional burden on Sponsors submitting clinical trial applications (CTA) and/or New Drug Submissions (NDS). A workshop initiated by CellCAN and BIOTECanada with participants from Environment and Climate Change Canada, Health Canada, the Public Health Agency of Canada and Innovation, Science and Economic Development (Ottawa, March 19, 2018) with invited stakeholders discussed approaches to streamline the environmental review process. The following main recommendations were the focus of the workshop:

1. A regulatory policy to clarify *Canadian Environmental Protection Act* (*CEPA*)'s definition of “living organism.” This is currently defined as “a substance that is an animate product of biotechnology.” A regulatory policy could potentially exempt “human cells touched by biotechnology for use in human medicinal products” from this definition to clarify any unintended overreach of *CEPA*, particularly as it applies to non-genetically modified cell therapies.
2. A guidance document to better interpret *NSNR(O)* Schedule 1 requirements by CTA/NDS sponsors to satisfy the environmental review process.
3. An amendment at the level of regulations, to the *NSNR (O)* to create a deferment to postpone environmental assessment of micro-organisms used in the manufacturing during investigational clinical trials (pre-market stage). The regulations would apply at the time of market authorization evaluation and review, when sufficient clinical data on vector shedding has been collected, as part of the investigational clinical trials.
4. Amendment to Schedule 4 of the *CEPA* to include the *Food and Drugs Act* and Regulations (*Food and Drugs Act /FDR*) as an exclusion to the application of CEPA. This would remove the current dual regulation of cell and gene therapies by both *CEPA* and *Food and Drugs Act* /*FDR*.

These recommendations and other options were discussed at the workshop. These recommendations if adopted will significantly streamline the current regulatory burden and harmonize environmental assessment requirements with other jurisdictions.

## Introduction

In Canada, gene therapies and oncolytic viruses for use as therapeutic agents to treat cancer are regulated as drugs under the *Food and Drugs Act* (R.S.C., 1985, c.F-27) (*Food and Drugs Act*) and associated regulations, including the *Food and Drug Regulations* (C.R.C., c.870). Oversight is provided primarily by Health Canada's Biologics and Genetic Therapies Directorate, which is responsible for regulation of biological drugs for human use based on sound evidence of the product's quality, safety, and efficacy.

In addition to regulation under the *Food and Drugs Act*, viral vectors for use in human gene therapy and oncolytic viruses are regulated as micro-organisms by Environment and Climate Change Canada (ECCC) under the *Canadian Environmental Protection Act* (*CEPA*) (S.C. 1999, c.33) and its associated regulation, the *New Substances Notification Regulations for (Organisms) (NSNR(O))*. *CEPA s. 3(1)* defines living organisms as “an animate product of biotechnology,” thereby including genetically-modified cells, and any cells (autologous or allogeneic) which are manufactured and processed by “biotechnology.”

The goal of the *NSNR(O)* is to ensure that no new micro-organism is introduced in Canada before its risks to the environment and human health have been assessed. However, the *NSNR(O)* include in its definition of micro-organisms, “virus-like particle or sub-viral particle”; this broad definition captures gene therapy vectors that are incapable of replication. The *NSNR(O)* apply to both import and Canadian manufacture of micro-organisms. Their research exemption does not apply to clinical research where the vector/virus is used in humans in a clinical setting outside of a contained laboratory.

Here we discuss the regulatory overlap and burden for developers of gene and cancer immuno- and viral therapies in Canada in complying with both Health Canada and ECCC regulatory requirements. Specifically, for clinical trials, developers must submit both a Clinical Trial Application (CTA) (which includes clinical and non-clinical data that speak to environmental impact) to Health Canada and information under *NSNR(O) Schedule 1* for an environmental risk assessment to ECCC. Currently the two reviews are not coordinated and the regulatory burden is not proportionate to the potential risks posed. The following recommendations aim to streamline the regulatory requirements and/or review processes aligned with other jurisdictions such as the United States.

Our recommendations were the focus of a stakeholder workshop organized by BIOTECanada and CellCAN and hosted by the Biologics and Genetics Therapeutics Directorate (BGTD). Attendees included representatives from Canada's biotechnology and pharmaceutical sectors, academic research institutions, and regulatory agencies, including Health Canada and ECCC. The workshop enabled participants to exchange views and discuss the pros and cons of various reform options. However, the recommendations presented here are the views of the authors and do not represent a consensus statement or the views of the regulatory agencies.

## Regulatory Context

**Figure 1:**
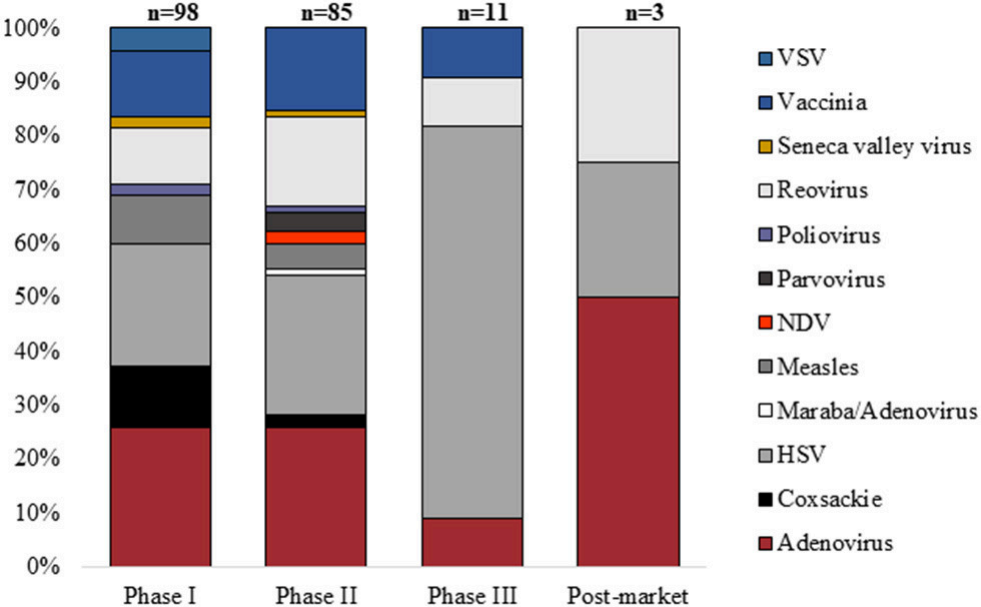
Proportion of oncolytic virus clinical trials (*n* = 217) registered by Phase between January 1999 and August 2018 using specific classes of virus (VSV, vesicular stomatitis virus; NDV, Newcastle disease virus; HSV, herpes simplex virus).

Clinical trials in gene therapy for cancer and other diseases advanced rapidly since the late 1990s (1, 2). The number of cancer cellular immunotherapy trials increased rapidly between 1995 (*n* = 7) and 2015 (*n* = 1,579), and the percentage of trials using genetically modified cells, such as chimeric antigen receptor T-cells (CAR-T), similarly increased between 2006 and 2015 (2). The majority of gene therapies are delivered to their *in vitro* or *ex vivo* target cell using a viral vector. One estimate for all gene therapy clinical trials suggests that over 1,800 clinical trials were completed or approved in 31 countries between 1989 and 2012 (3). The most common viral vectors are adenovirus, retrovirus, naked/plasmid DNA, vaccinia virus, poxvirus, adeno-associated virus, herpes simplex virus, and lentivirus (1). In addition, between 1999 and 2018, 217 oncolytic virus clinical trials were registered worldwide for a wide range of cancers (Bubela et al. unpublished data; Figure 1).

Not only has clinical trial activity increased, but national health and safety regulators have approved some gene therapies for use in humans, and these must now be considered for coverage by public and/or private payers before they can be accessed by patients. In 2017, the United States (US) Food and Drug Administration approved two CAR-T cell therapies: Novartis' Kymriah (4) and Gilead's Yescarta (5) for patients up to 25 years of age with B-cell precursor acute lymphoblastic leukemia (ALL) that is refractory or in second or later relapse and for the treatment of adult patients with relapsed or refractory large B-cell lymphoma, respectively. Kymriah™ was also approved by Health Canada in the Fall of 2018 for patients from 3 to 25 years of age with refractory B-cell acute lymphoblastic leukemia (ALL) and adult patients with relapsed or refractory large B-cell lymphoma (6). Approvals were also granted in the European Union (7, 8). In addition, the Food and Drug Administration has also recently approved Spark Therapeutics' LUXTURNA™ for treatment of the X-linked retinal dystrophy, Leber congenital amaurosis (9). The EMA has similarly approved three gene therapies including LUXTURNA™, UniQure's Glybera for a rare metabolic disease (now withdrawn from use due to market failure) (10) and GlaxoSmithKline's Strimvelis for a rare immune deficiency in children (11). Collectively, these approvals raise expectations among Canadian patients for access to gene and cell therapies.

### Canada Is Advancing Clinical Trials

Between 1989 to 2012, 24 gene therapy trials were conducted in Canada, and 14 cellular immunotherapy trials between 1995 and 2015 (2). More clinical trials are planned. In addition, there have been 23 clinical trials of oncolytic virus therapies in Canada between 1999 and 2018 (Bubela et al. unpublished data), primarily in Ontario, Alberta and Quebec. While some trials were sponsored by companies or organizations outside of Canada, others were developed in Canada, such as trials for reovirus and vaccinia for a range of cancers. Most recently, Turnstone Biologics Inc. is testing a maraba/adenovirus combination in non-small cell lung cancer (NCT02879760)^1^, and BioCanRx announced the funding of the first made-in-Canada CAR-T cell trial with involvement of several centers across the country for vector production, T cell transfection and product manufacturing and patient treatment (https://biocanrx.com/wp-content/uploads/2017/02/Enabling-3-HOLT-dashboard3.pdf).

### Canadian Organizations Promote the Clinical Translation of Gene and Cell Therapies

CellCAN is a pan-Canadian non-profit organization established in 2014 that is part of the Government of Canada's Networks of Centers of Excellence. CellCAN brings together Canada's leading cell manufacturing centers (manufacturing product characterization, bioengineering, ethical and legal regulatory policy) to improve the quality, safety and feasibility of cell and gene therapy in Canada through optimal manufacturing practices^2^.

BIOTECanada is the national industry association with over 200 members located nationwide, reflecting the diverse nature of Canada's health, industrial and agricultural biotechnology sectors. In addition to providing significant health benefits for Canadians, the biotechnology industry has quickly become an essential part of the transformation of many traditional cornerstones of the Canadian economy including manufacturing, automotive, energy, aerospace and forestry industries^3^.

In addition, Canada's Networks of Centers of Excellence program has supported two NCEs relevant to cell and gene therapies. It has supported the Stem Cell Network^4^ since 2001 and, BioCanRx, Canada's Immunotherapy Network, to focus on translational strategies for rational combination immunotherapies in addition to immunotherapies used alone, since 2015^5^. Both networks support policy and regulatory development, in addition to advancing research into partner-funded clinical trials.

The Canadian research community imports vectors from international sources and manufactures its own vectors for use in clinical trials. Endogenous manufacture is likely to increase as clinical trial facilities begin operation in Montreal, Toronto, Ottawa, Edmonton, and Victoria. Federal and provincial investments in cell and gene therapy clinical trial facilities and partnerships are evidence of support for advancing clinical trials as Canada moves toward an innovation-based economy.

## Assessment of Policy/Guidelines Options and Implications

### Regulation of Gene Therapy and Oncolytic Virus Products

In light of the clinical trial and therapeutic landscape for gene therapy and oncolytic virus products in Canada, it is timely to consider the intersection of regulatory oversight between Health Canada and ECCC. The intersection derives from New Substance Notification described in the Canadian Environmental Protection Act. Such release could occur directly, such as through spillage or improper disposal of medical waste, or indirectly, by way of viral shedding from a treated patient. In the following section, we first discuss the current regulatory environment in Canada, contextualizing it with those of similar countries/regions, and then discuss a proportionate regulatory response for Canada relative to the risks posed by these products. We start with a discussion of Health Canada oversight of “environmental release” via viral shedding, followed by a discussion of ECCC's oversight.

### Health Canada Regulates Gene and Cell Therapy Guidance as Drugs Under the Food and Drugs Act and Regulations Food and Drugs Act/FDR)

However, a Guidance document “Preparation of Clinical Trial Applications for use of Cell Therapy Products in Humans” is available (12). Under the *FDR*, an application for a drug establishment license (under Division 1A), or an application for a clinical trial application for drugs, including cell and gene therapies (under Division 5) or an application for market authorization for drugs (under Division 8) would trigger an environmental risk assessment by ECCC.

Health Canada is a member of the International Conference on Harmonization (ICH) Steering Committee. It refers clinical trial sponsors to the ICH Technical Requirements for Registration of Pharmaceutical for Human Use (ICH) guidelines, which include those for safety testing of vectors (13), and three consideration documents on general principles to address viral/vector shedding (13), inadvertent germline integration (14), and oncolytic viruses (15). Health Canada may require viral/vector shedding studies be conducted as part of clinical trials.

The US Food and Drug Administration developed a guidance document for industry on “*Design and Analysis of Shedding Studies for Virus or Bacteria-Based Gene Therapy and Oncolytic Products”*(16). The Guidance Document defines “shedding” as release of virus or bacteria-based gene therapy products (VBGT products) or oncolytic products from the patient through one or all of the following ways: excreta (feces); secreta (urine, saliva, nasopharyngeal fluids, etc.); or through the skin (pustules, sores, wounds). Shedding raises the possibility of transmission from treated to untreated individuals.

The US Food and Drug Administration recognizes that “in most cases, the potential for transmission to untreated individuals is extremely low when VBGT or oncolytic products are shed because the derivation methods and modifications made during product development lead to attenuation” (16). However, since the theoretical risk of infection remains, shedding studies may be appropriate prior to licensure, and these are typically carried out as part of safety or efficacy clinical trials, not as stand-alone studies. Shedding studies may be pre-clinical animal studies or human studies. Their design depends on biological characteristics (replication competence, immunogenicity, persistence and latency, tropism, and/or stability of product attenuation).

Shedding data in clinical studies “provides a shedding profile of a product in the target patient population and is used to estimate the potential of transmission to untreated individuals” (16). The guidance document provides information on the trial phase in which to collect data, depending on the attenuated state of the VBGT or oncolytic product; shedding study design; the analytical assays to measure shedding; the analysis of shedding data; and reporting requirements for shedding studies.

The ICH Technical Requirements for Registration of Pharmaceuticals for Human Use similarly provide *General Principles to Address Virus and Vector Shedding* (13). They provide “recommendations for designing non-clinical and clinical shedding studies when appropriate. In particular, emphasis will be on the analytical assays used for detection, and considerations for the sampling profiles and schedules in both non-clinical and clinical studies.”

The purpose of the Canadian Environmental Protection Act (CEPA) is to contribute to “sustainable development through pollution prevention” (17). While it enables the making of regulations, the CEPA is not supposed to overlap with other regulations. Specifically, it states that it has the authority to “make an order declaring that the provisions of the regulation do not apply in an area under the jurisdiction of the government” [CEPA, s. 10(3)].

Another aspect of *CEPA* that is contentious is its broad definition of living organisms. Part 6 of the *CEPA* states that “ *living organisms* “means a substance that is an animate product of biotechnology,” a definition that includes “*any distinguishable kind of organic or inorganic matter, whether animate or inanimate”* and “*significant new activity* includes, in respect of a living organism, any activity that results or may result in (a) the *entry or release of the living organism into the environment in a quantity or concentration* that, in the Ministers' opinion, is *significantly greater than the quantity or concentration of the living organism that previously entered or was released into the environment”* (*CEPA*, s.104, emphasis added). The implication is that *CEPA* is primarily concerned with animate products of biotechnology that are introduced in a significant quantity or concentration to pose a threat to the environment.

The *CEPA* applies to *living organisms* that are manufactured in or imported into Canada. The process for approval places the burden on the Minster to add living organisms to the Domestic Substances List within 120 days [*CEPA*, s. 112] once s/he has been provided with all required and/or additional information/tests as specified under the *CEPA*. However, the intent of the *CEPA* is not to regulate if equivalent provisions exist. This should be the case for the environmental impact assessment conducted under the CTA according to the *Food and Drug Act/FDR*. Schedule 4 names the Acts and Regulations that currently meet the requirements: *Pest Control Products Act (Pest Control Products Regulations); Seeds Act (Seeds Regulations); Fertilizers Act (Fertilizers Regulations); Feeds Act (Feeds Regulations); Health of Animals Act (Health of Animals Regulations)*. The Schedule was last amended in 2001 and does not include the *Food and Drug Act /FDR*.

### New Substances Notification Regulations (Organisms)

NSNR(O) regulations are created under s. 114 of the CEPA (18). The CEPA does not define micro-organism, it only defines living organism. Viruses, especially those that are non-replicating, are not considered living organisms under a scientific/taxonomic definition of the term. However, the regulations may “prescribe conditions and circumstances in respect of a living organism in terms of (a) whether or not the living organisms is a member of a group of living organisms established by regulations… [s. 114(3)]. The *NSNR(O)*, therefore, define and regulate “micro-organism,” under the scope of a “microscopic organism that is… (b) a virus, virus-like particle or sub-viral particle.” This broad definition therefore moves beyond animate organisms as defined under the *CEPA* to encompass viral vectors used for gene therapy, including those that are non-replicating. However, like the *CEPA*, the intent of the *NSNR(O)* is not to regulate if equivalent provisions exist. The NSNR(O) regulations (s.2) “do not apply in respect of an organism that is manufactured or imported for use that is regulated under any other Act or regulations listed in Schedule 4 to the [*CEPA*].” However, as stated above, no Acts or Regulations under the jurisdiction of Health Canada or the Public Health Agency of Canada are listed in Schedule 4 of *CEPA* (19). This creates an indeterminate basis for regulating non-replicating viral vectors, virus-like particles and sub-viral particles that is currently deemed more appropriate to trigger the NSNR(O).

Pre-clinical research and development of viruses and viral vectors for gene therapy and oncolytic viruses are exempt from *NSNR(O)* [s. 2(3)], because they are used for research and development in a contained facility and are generally manufactured or imported in quantities below the minimum required for *NSNR(O)* regulation, which vary, depending on the level of the organism as identified in the Canadian Laboratory Biosafety Guidelines (20). However, vectors and oncolytic viruses for use in clinical trials and approved drugs are both administered to patients, generally in a hospital or clinical setting, and therefore fall outside the contained facility exemption in the regulations.

It is apparent that the *NSNR(O)* have been interpreted to apply because of the theoretical risk that the vector/virus could be shed into the environment following administration into patients. As noted above, shedding is already monitored by Health Canada, under the existing *FDR*. In addition, the early-stage of clinical trials means that micro-liters of vector are administered to a small number of patients (6–12 participants for Phase 1 trials). This volume does not fall within the definition of environmental introduction of significant quantity or concentration as contemplated by the *CEPA*.

### Regulation of Importation of Viruses and Viral Vectors Under Other Acts

Depending on the type of virus or viral vector, importation may further require a license from the Public Health Agency of Canada under the *Human Pathogens and Toxins Act*. That Act no longer applies for a drug in dosage form and whose sale is permitted under the *Food and Drugs Act*, however, it would apply to experimental drugs that contain a human pathogen or toxin that has not yet received regulatory approval. In its assessment, the Public Health Agency of Canada considers the levels of risk posed by the human pathogen/toxin and regulates according to risk group for human pathogens.

Note also that some oncolytic viruses may be regulated under the *Health of Animals Act* and are therefore exempt from the *NSNR(O)*. The exception is due to additional regulatory scrutiny that is generally required to address the additional risks of environmental release and epidemic spread in domestic animals, particularly as they relate to agricultural livestock and they are regulated under agricultural regulations (Agriculture and Agri-food Canada) and therefore they exempt from NSNR (O) regulations, others are not, and therefore are captured under NSNR (O). This leads to the following regulatory complexity for proposed combination clinical trials: the trial (and potential shedding) is regulated by Health Canada but one virus is regulated under the *Health of Animals Act* and the second adjuvant virus is regulated under the *NSNR(O)*.

The impact of the *NSNR(O)* on developers of clinical-grade viral vectors for gene therapy or oncolytic viruses is a 120 days delay while the information package is reviewed (s. 5). Note that the 120 days may be longer if the information package is deemed to be incomplete, because the 120 days statutory review timeline applies only once the information package is complete. This is incongruent with the current 30 days default review of Clinical Trial Applications (CTA) by Health Canada. For all Phase I-III clinical trials, sponsors are required to submit a CTA for clinical and quality review. Health Canada issues a no-objection” letter (NOL) if there are no outstanding issues allowing the trial to commence, conditional on institutional ethics approvals. Typically, Health Canada seeks clarification on elements of the application (Clarifax); sponsors need to respond to Clarifax inquires within a 2 days period. If the deficiencies are not properly clarified or are too egregious, sponsors may withdraw the CTA or risk a Non-Satisfactory Notice (NSN). The rapid 30 days turnaround or default approval is valued by sponsors and is aligned with the US Food and Drugs Administration review process of investigational new drug (IND) submissions (21).

The 120 days independent review by ECCC thus adds substantially to the 30 days review by Health Canada for CTA submissions. Additionally, there is no approved guidance document for developers on the information package required relevant to clinical-grade viral vectors for gene therapy or oncolytic viruses to be used in clinical trials, although ECCC has been developing a draft guidance for this purpose, beyond what currently exists (22). The extensive information required in respect of micro-organisms is outlined in Schedule 1 of *NSNR (O)* (18) and includes information on taxonomy, infectivity, pathogenicity to non-humans, resistance to antibiotics, resistance to metal ions, toxigenicity, and mechanism of dispersal and interaction with dispersal agents. However, many viral vectors are non-infectious, non-replicating, viral sub-particles (not intact viruses) therefore most of the evidence requirements under Schedule 1 are not applicable.

The environmental risk assessment once conducted *and* approved allows listing of medicinal ingredients in human and veterinary prescription and non-prescription drugs to be listed on the Domestic Substance List (DSL). The DSL under *CEPA 1999* lists all medicinal ingredients in human and veterinary prescription and non-prescription drugs being imported into, manufactured in, or used in Canada. However, while many gene therapies will utilize the same viral-vector backbone (e.g., AAV2), a new application will be required for each gene therapy developed to target different diseases, or for minor modifications made to the vector construct. Although ECCC allows for consolidating or matching such applications, there is no guidance on how this will be practically implemented, creating uncertainty.

## Canada's Regulatory Process Compared to Other Jurisdictions

While other jurisdictions also regulate for both environmental and health risks, they typically use a single review process for gene therapies and oncolytic viral products. The US Food and Drug Administration conducts environmental assessments as part of a single review process for investigational new drugs. Sponsors do not duplicate the submissions or deal with external agencies for the environmental review.

In its review of advanced medicinal therapy products (ATMPs), the European Medicines Agency (EMA) requires completion of an environmental risk assessment (ERA) (23) as *part of the dossier* for evaluation for market authorization as specified in Directive 2001/18/EC (24). The ERA is based on pre-clinical and/or clinical data, which includes shedding studies, data on the potential hazards posed by the genetically modified organism including stability, pathogenicity, attenuating modifications, replication competence, altered susceptibility to the immune system, altered tropism, unintended transfer. These data constitute the ERA, together with the likelihood of the adverse event occurring and the consequences of the adverse event. Additionally, ERA are also conducted on genetically modified organisms (GMO) including gene therapies and gene-modified cell therapies at the clinical trial stage. However, different member states of the EU have different requirements for the ERA; some consider classify clinical trials as deliberate release, while others consider it contained release resulting in wide variability in how the risk is assessed (24, 25).

In Japan, there is *joint* review by the Ministry of Environment and Ministry of Labor, Health and Welfare (MLHW) for use of Living Modified Organisms [LMO, based on the international agreement *Cartagena Protocol on Biosafety to the Convention on Biological Diversity* (26)]. In Japan, there are two types of uses of LMOs: Type 1 use, which does not have any preventative measures against dispersal into the environment and Type 2 use, which includes production, handling, transport under containment measures. Viral vectors are classified as Type 1 LMOs and require approval from Ministry of Environment and MLHW, who consult with the Food and Pharmaceuticals Affair Council in approving the *Biological Diversity Risk Assessment* reports provided by the sponsors.

In contrast, Canada imposes *separate and independent* regulatory oversight under environmental protection laws at the time of both clinical investigation and during new drug submission reviews. These laws were developed in the 1990s, prior to recent advances in gene therapy and immunotherapy clinical trials. In light of expected increases in the manufacture and import of these products for clinical testing in Canada, it is timely to re-consider the additional regulatory burden imposed on Canadian developers compared to other jurisdictions.

## Actionable Recommendations and Conclusions

The recommendations discussed at the workshop vary in their potential impact, from broad regulatory impact to narrow regulatory impact. They also vary in their procedural complexity for implementation. For example, changes to the Acts require Parliamentary intervention, while reforms to the regulations require adherence to a consultation and reform process. We list the recommendations below from broadest to narrowest impact, reflecting long-term goals and reforms that could be implemented in the short-term.

### Recommendation 1

Amend Schedule 4 of the *CEPA* to include the Food and Drugs Act*/FDR* (or relevant sections thereof) as an exclusion to the application of *CEPA*. Health Canada already requires shedding studies as part of environmental safety monitoring and references international guidelines for sponsors of clinical investigations of advanced medicinal products. The *NSNR(O)* are duplicative of this oversight process, which was not the intent. Indeed, inclusion in Schedule 4 includes other acts and regulations to exempt redundant review, where equivalent provisions exist. An amendment to *CEPA* would capture all substances currently dually regulated by both *CEPA* and the Food and Drugs Act, and would remove CEPA's application with respect to NSNR(O) requirements for new micro-organisms.

In parallel, amendments to the *FDA/FDR/NSNR(O)* would likely be required. In particular, the *FDR* (Part C, Division 5) and related guidance documents may need to be amended to include environmental reporting and assessment requirements to provide sufficient environmental oversight. Indeed, an Environmental Impact Initiative (EII), led by the Health Product and Food Branch at Health Canada, is currently evaluating how to make such amendments and their legal implications and is evaluating a staged approach at time of importation, at the time of CTA submission and imposing the largest burden in terms of compliance to Schedule 1 of *NSNR (O)* at the time of market authorization. The EII is also considering how to integrate the environmental risk assessment with Health Canada submission stages, creating a single-window for submitting data; this would align the environmental review process for CTAs with the process at the *Food and Drugs Act* for investigational new drugs (INDs). However, this approach necessitates a legal decision by Justice Canada that the mandate of *FDR* under *FDA* is equivalent to *CEPA* with respect to environmental regulation.

### Recommendation 2

Amend the definition of “micro-organism” in s. 1 of the *NSNR(O)* so that the definition no longer includes a “virus-like particle or sub-viral particle,” especially those used in human medicinal products. This recommendation would not impact current review for oncolytic viruses, but would exempt other cancer immunotherapies that are manufactured using viral vectors.

### Recommendation 3

Amend or clarify *CEPA*'s definition of “living organism” as “an animate product of biotechnology” under s. 3(1) of CEPA. The current definition applies to all cell therapy products including autologous products, which was not the intent of *CEPA*, and accordingly constitutes regulatory over-reach. Regulatory policy should clarify this definition in *CEPA* to streamline the review process for cell and gene therapies.

### Recommendation 4

Amend the *NSNR(O)* such that their provisions would not apply to micro-organisms imported for or used in manufacturing during the pre-market investigational stage. The regulations would apply at the time of market authorization when sufficient clinical data on vector shedding had been collected and manufacturing volumes would be expected to increase. This option poses a judicial and efficient use of regulator resources, however, a consultation process would be needed to initiate such a policy change.

### Recommendation 5

In the interim, we recommend that ECCC work with Health Canada to provide clinical trial sponsors with guidance on *NSNR(O)* Schedule 1, including the sections not relevant for viral vectors that are non-infectious and non-replicating. Alternatively, these agencies could make the process for obtaining administrative waivers more transparent. ECCC has been responsive to this request and has provided a draft guidance document for input and comment prior to finalization.

## Conclusion

The improvement of the regulatory process and reduction in regulatory overlap between *CEPA* and the Food and Drugs Act and related regulations will facilitate the manufacturing, clinical trials, and therapeutic use of gene, cell and viral therapies in Canada in a timely manner. This is especially true as new immunotherapies are being developed to treat previously intractable forms of cancer and other diseases, potentially providing novel healthcare solutions for Canadians. A workshop held by Health Canada, ECCC, BIOTECanada, and CellCAN articulated the regulatory burdens and proposed several solutions based on existing frameworks to simplify and streamline this process. These proposed solutions are a first step in this process of instituting regulatory reform and will need continuous engagement and support from multiple stakeholders to result in real reform. The EII initiative spearheaded by Health Canada and the proposal for new guidelines to meet Schedule 1 requirements of *NSNR (O)* by ECCC are examples of initiatives taken by regulators who are invested in transparent, streamlined and proportionate regulations that will advance cell and gene therapy translational research in Canada, while safeguarding Canadians and the environment.

As next steps, a follow-up workshop will be initiated between the various stakeholders including Health Canada and ECCC, the clinical and research community and industry partners to focus on the recommendations that gained the most traction. To address the full scope of recommendations, an economic evaluation may serve to detail the impact of the current NSNR(O), thus providing a driving force to create the necessary exemption for human health products, and especially autologous therapies.

## Data Availability

All datasets generated for this study are included in the manuscript and/or the supplementary files.

## Author Contributions

TB and SV worked on the concept, the edits and the final copy of this article. RB co-organized the workshop with BIOTECanada and coordinated the workshop discussions with the stakeholders, Health Canada and Environment and Climate Change Canada.

### Conflict of Interest Statement

SV has a regulatory consulting company which did not conflict with the preparation of this manuscript. RB is Vice President, Biotechnology and Industry Affairs at BIOTECanada, the national industry association for Canada's health, industrial and agricultural biotechnology sectors. The remaining author declares that the research was conducted in the absence of any commercial or financial relationships that could be construed as a potential conflict of interest.

## Footnotes

^1^https://clinicaltrials.gov/

^2^www.cellcan.com

^3^www.biotech.ca

^4^stemcellnetwork.ca

^5^biocanrx.com
